# Vitamin D receptor signaling improves Hutchinson-Gilford progeria syndrome cellular phenotypes

**DOI:** 10.18632/oncotarget.9065

**Published:** 2016-04-27

**Authors:** Ray Kreienkamp, Monica Croke, Martin A. Neumann, Gonzalo Bedia-Diaz, Simona Graziano, Adriana Dusso, Dale Dorsett, Carsten Carlberg, Susana Gonzalo

**Affiliations:** ^1^ Edward A. Doisy Department of Biochemistry and Molecular Biology, St Louis University School of Medicine, St. Louis, MO, USA; ^2^ School of Medicine, Institute of Biomedicine, University of Eastern Finland, Kuopio, Finland; ^3^ Bone and Mineral Research Unit, Hospital Universitario Central de Asturias, Oviedo, Spain

**Keywords:** DNA repair, genomic instability, laminopathies, progeria, vitamin D receptor, Gerotarget

## Abstract

Hutchinson-Gilford Progeria Syndrome (HGPS) is a devastating incurable premature aging disease caused by accumulation of progerin, a toxic lamin A mutant protein. HGPS patient-derived cells exhibit nuclear morphological abnormalities, altered signaling pathways, genomic instability, and premature senescence. Here we uncover new molecular mechanisms contributing to cellular decline in progeria. We demonstrate that HGPS cells reduce expression of vitamin D receptor (VDR) and DNA repair factors BRCA1 and 53BP1 with progerin accumulation, and that reconstituting VDR signaling via 1α,25-dihydroxyvitamin D_3_ (1,25D) treatment improves HGPS phenotypes, including nuclear morphological abnormalities, DNA repair defects, and premature senescence. Importantly, we discovered that the 1,25D/VDR axis regulates *LMNA* gene expression, as well as expression of DNA repair factors. 1,25D dramatically reduces progerin production in HGPS cells, while stabilizing BRCA1 and 53BP1, two key factors for genome integrity. Vitamin D/VDR axis emerges as a new target for treatment of HGPS and potentially other lamin-related diseases exhibiting VDR deficiency and genomic instability. Because progerin expression increases with age, maintaining vitamin D/VDR signaling could keep the levels of progerin in check during physiological aging.

## INTRODUCTION

HGPS is a rare but severe premature aging disease. Affected patients die in their teens from myocardial infarction or stroke as a result of rapidly progressive atherosclerosis [1–3]. Most cases are caused by a G608G(GGC > GGT) single-base mutation within exon 11 of the *LMNA* gene [4, 5], which codes for lamin A/C *via* alternative splicing. This mutation activates a cryptic splice site that prevents proper processing of prelamin A to mature lamin A and produces a permanently farnesylated and carboxymethylated toxic product called “progerin”. These post-translational progerin modifications seem to play a major role in the pathophysiology of disease [6–9]. Progerin disrupts the nuclear lamina, a compartment essential for nuclear structure and function, provoking an array of cellular aberrations, including nuclear morphological abnormalities, increased DNA damage and genomic instability, epigenetic alterations, and disrupted cell signaling, all of which ultimately cause premature senescence [10–16].

Therapies for this fatal disease are desperately needed. The combination of farnesyltransferase inhibitors (FTIs), statins and bisphosphonates, which reduce prenylation of progerin, are the best treatment strategy currently available to physicians, but only extend life by 1.6 years on average [17, 18]. Further, FTIs can induce a variety of noxious side effects. Thus, there is a tremendous need for new treatments with fewer side effects. Interestingly, progerin accumulation is also observed in old individuals, suggesting its participation in the normal aging process [19]. Identifying therapeutic strategies that reduce the toxic levels of progerin could have applicability for HGPS patients and for the general aging population.

Vitamin D is essential for proper calcium and phosphate homeostasis, *via* its biologically most active metabolite 1,25D. Most genomic actions of vitamin D are mediated by the vitamin D receptor (VDR), being the only high-affinity nuclear receptor for 1,25D [20]. However, expression of VDR in tissues that are not associated with calcium and bone homeostasis suggested that the vitamin D/VDR axis could exert other functions. Alterations in the vitamin D/VDR status have considerable effects at the cellular and organismal level and contribute to a wide variety of diseases [21, 22]. Interestingly, VDR knockout mice develop a premature aging phenotype similar to HGPS patients, with early alopecia, growth retardation, muscle atrophy, cardiovascular disease, and reduced lifespan [21, 23]. Additionally, vitamin D/VDR signaling is important for protecting against atherosclerosis [24, 25], the pathology ultimately underlying death in HGPS patients. Consequently, we investigated whether the vitamin D/VDR axis could have an impact on the phenotype of HGPS patient-derived cells.

Here, we demonstrate for the first time that proper lamina organization is required for appropriate VDR function and that HGPS patient cells become VDR deficient upon passage in culture. Importantly, 1,25D supplementation, which counteracts VDR loss, improves many of the phenotypes of HGPS cells, including nuclear morphology and unrepaired DNA damage while delaying the onset of senescence. Most significantly, we demonstrate that prolonged 1,25D treatment leads to a dramatic decrease of progerin levels. These results advance targeting the vitamin D/VDR axis as a potential therapeutic strategy for improving HGPS patient health.

## RESULTS

### Alterations in the nuclear lamina cause VDR deficiency

**Figure 1:**
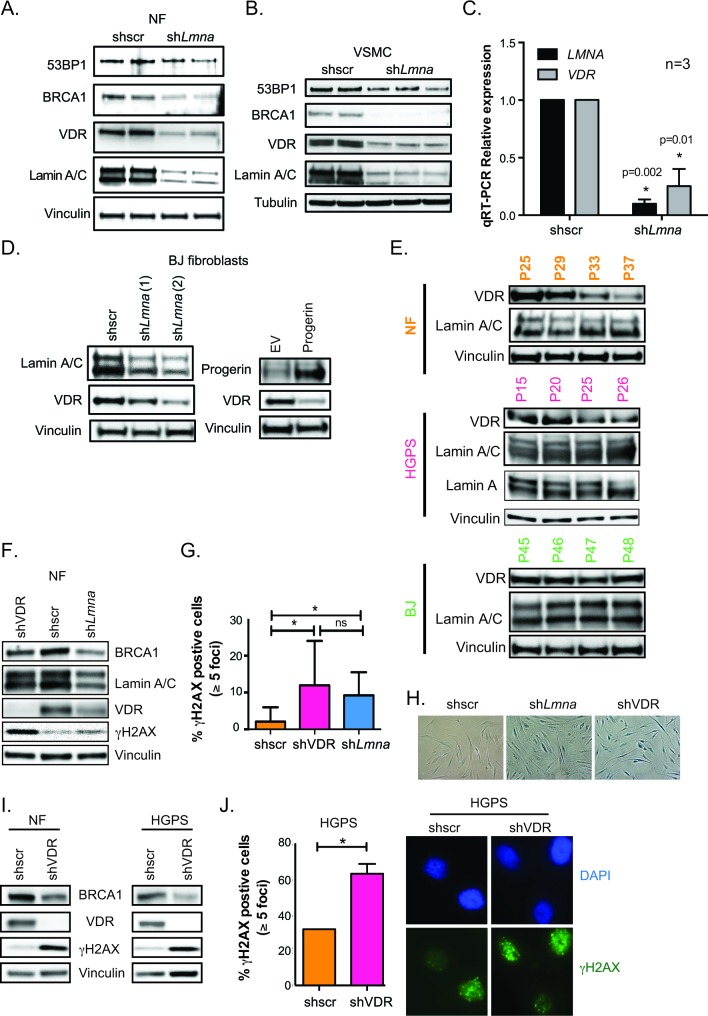
VDR deficiency in cells with disrupted nuclear lamina **A.** Human NF lentivirally transduced with shRNA targeting lamin A/C (shLmna) or scrambled (shscr), and processed for immunoblotting (2 independent transductions). Vinculin is loading control. **B.** The same experiments as in (A) performed in human VSMC. Tubulin is loading control. **C.** Relative expression of *LMNA* and *VDR* transcripts by qRT-PCR in VSMC after depletion of lamin A/C or VDR. Results are mean±sem of 3 biological repeats. **D.** BJ fibroblasts were lentivirally transduced with 2 independent shRNAs targeting lamin A/C (left panels) or retrovirally transduced with progerin (right panels) and processed for immunoblotting. **E.** Different human fibroblasts (BJ, NF and HGPS) were collected at increasing passages in culture to monitor levels of VDR and lamin A/C by western blot. **F.** NF were lentivirally transduced with shscr, shLmna, or shRNA targeting VDR (shVDR) and processed for immunoblotting. **G.** Immunofluorescence (IF) with γH2AX antibody in NF depleted of VDR, lamin A/C, and control. Quantitated percentage of cells with more than 5 γH2AX foci. Graph represents mean±sem of 3 independent experiments. **H.** Images show how depletion of lamin A/C or VDR leads to accumulation of β-galactosidase positive cells, a marker of senescence (after 2 weeks). **I.** NF and HGPS cells were depleted of VDR and levels of BRCA1, VDR, and γH2AX monitored by immunoblotting. **J.** IF performed in HGPS depleted of VDR (shVDR) and control (shRNA) and percentage of γH2AX-positive cells quantitated. Images of IF showing accumulation of γH2AX in HGPS cells depleted of VDR. *p value of statistical significance (*p ≤ 0.05).

The nuclear lamina is a critical scaffold for many transcription factors. In lamin A/C-deficient cells, major changes occur in gene signaling and transcription factor localization [26, 27]. Because VDR is an important transcription factor and can accumulate at the nuclear envelope [28], we tested whether disruptions in the nuclear lamina impact VDR levels. We discovered that lamin A/C depletion *via* lentiviral transduction with specific shRNAs results in a marked decrease in VDR levels in human primary normal fibroblasts derived from parents of HGPS patients (referred to as NF) (Figure 1A). Lamin A/C loss correlates with decreases in BRCA1, and to a lesser extent in 53BP1 levels, key factors in DNA repair by homologous recombination (HR) and non-homologous end joining (NHEJ) respectively, as previously observed in mouse fibroblasts and breast cancer cells [29]. Similar decreases in VDR and DNA repair factors were observed upon depletion of lamin A/C in human primary vascular smooth muscle cells (VSMC) (Figure 1B), a cell type particularly susceptible to alterations in lamin A/C function [30, 31]. In addition, quantitative RT-PCR (qRT-PCR) revealed a profound decrease in VDR transcript levels following lamin A/C depletion (Figure 1C). The reduction in VDR was confirmed in foreskin fibroblasts (BJ) transduced with two independent shRNAs targeting lamin A/C, ruling out that off-target effects are responsible for VDR loss (Figure 1D). Interestingly, disruption of the nuclear lamina by ectopic expression of progerin in these cells also results in down-regulation of VDR (Figure 1D). Importantly, VDR levels decrease during proliferation of HGPS patient-derived fibroblasts in culture (Figure 1E), as well as during proliferation of normal fibroblasts isolated from the parent of the patient. However, while the VDR decrease in HGPS cells is evident by passage 25, the VDR decrease in NF is not observed until much later passages. In contrast, BJ fibroblasts did not show a decrease in VDR at the passages tested, suggesting differences in VDR expression during proliferation among different lines of human primary fibroblasts. Of note, the decrease in VDR levels was detected in proliferating cells, prior to senescence. Altogether, these data demonstrate that disruption of the nuclear lamina, either by lamin A/C loss or progerin overexpression, results in reduced VDR expression in primary human cells.

### VDR depletion causes DNA damage in normal and HGPS cells

Signaling through the vitamin D/VDR axis regulates indirectly the expression of BRCA1 in some contexts [32, 33]. Given that NF and HGPS cells grown in culture exhibit reduced VDR levels (Figure 1E), we determined if VDR loss could contribute to DNA repair defects in these cells. Consistent with this notion, depletion of VDR in NF leads to decreased BRCA1 levels and accumulation of DNA damage, as shown by elevated γH2AX levels, mirroring the phenotype of lamin A/C-depleted cells (Figure 1F). These changes were cell cycle independent, as shown by the similar cell cycle profiles between VDR-proficient and VDR-deficient NF at the time of sample collection for protein analysis (Figure S2A). DNA damage accumulation upon VDR depletion in NF was confirmed by immunofluorescence, showing a marked increase in the percentage of γH2AX-positive cells (Figure 1G). Importantly, VDR depletion eventually led to a growth arrest with characteristics of senescence, as shown by positivity for β-galactosidase (Figure 1H), recapitulating the phenotype of lamin A/C-depleted cells (Figure 1H), BRCA1-depleted cells [34], and BJ fibroblasts depleted of VDR [33]. Importantly, the effect of VDR loss causing BRCA1 reduction and accumulation of DNA damage was confirmed in a second line of NF (Figure 1I) and in HGPS patient-derived fibroblasts of early passage (Figure 1I and 1J). These data uncover a role for VDR in the maintenance of DNA repair. VDR loss leads to BRCA1 down-regulation, accumulation of unrepaired DNA damage, and entry into senescence, suggesting that the DNA repair defects observed in cells with a disrupted nuclear lamina could be exacerbated by VDR deficiency.

### 1,25D treatment ameliorates DNA repair defects in HGPS cells

**Figure 2:**
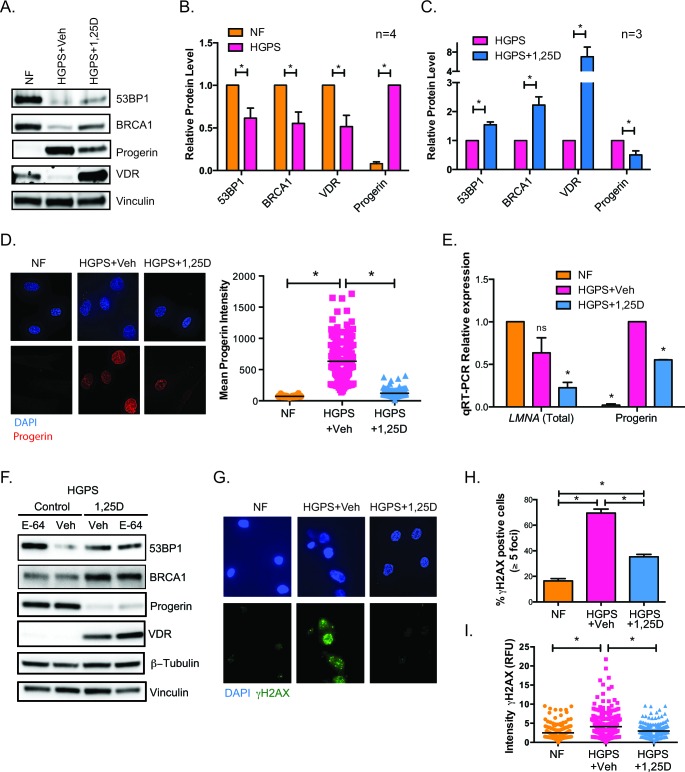
Phenotypes of HGPS cells are rescued by vitamin D **A.** HGPS cells were grown in culture with 1,25D (10^−7^M) or vehicle and collected for western blot prior to entering senescence (passage 25). Levels of DNA repair factors 53BP1 and BRCA1, VDR, and progerin were compared to NFs. **B.** Densitometry of immunoblots comparing levels of 53BP1, BRCA1, VDR, and progerin between NF and HGPS cells (mean±sem of 4 biological repeats). **C.** Densitometry as in (B) comparing HGPS cells under prolonged treatment with 1,25D or vehicle as control (mean±sem of 3 biological repeats). **D.** DAPI and IF staining of progerin shows that accumulation of progerin in HGPS cells is reduced by prolonged 1,25D treatment (passage 27). Graph shows quantitation of progerin labeling intensity (relative fluorescence units) in NFs and in HGPS cells subjected to prolonged treatment with vehicle or 1,25D. DAPI staining was used to demarcate nuclei and intensity of progerin labeling measured using ImageJ program. A total of 200 cells were quantitated in each condition. **E.** HGPS fibroblasts were grown in culture for at least 90 days with 1,25D or vehicle, and qRT-PCR performed to monitor levels of total *LMNA* and progerin transcripts (passage 29). NF of similar passage were used as control. Results are the mean±sem of 3 independent experiments. **F.** Fibroblasts derived from a second HGPS patient were subjected to prolonged 1,25D treatment or vehicle control (passage 27). These two lines were treated with the cathepsin inhibitor E64 or vehicle for 24 h, and samples processed for immunoblotting. **G.** DAPI and IF staining of γH2AX shows that accumulation of DNA damage in HGPS cells is reduced by prolonged 1,25D treatment. **H.** Quantitation of percentage of cells positive for γH2AX in 3 biological repeats. **I.** Quantitation of γH2AX labeling intensity (relative fluorescence units) in NF and in HGPS cells under prolonged 1,25D treatment. DAPI staining was used to demarcate nuclei and intensity of γH2AX labeling measured using ImageJ program. A total of 200 cells were quantitated. *p value of statistical significance (*p ≤ 0.05).

HGPS cells in culture progressively accumulate progerin [10], which in turn causes nuclear deformation and fragility, accumulation of DNA damage, and altered signaling pathways [11, 16, 35]. These defects eventually cause premature entry into senescence. Given the association between VDR loss and DNA repair defects, we tested if activation of VDR signaling by 1,25D treatment ameliorates the phenotypes of HGPS patient-derived fibroblasts. We performed prolonged treatment with 1,25D or vehicle as control, because the abnormalities of HGPS cells are exacerbated with time in culture. In addition, the prolonged treatment allowed us to determine if 1,25D impacts premature entry into senescence. HGPS cells media was supplemented with 1,25D (10^−7^M) every 3 days for 90 days, since 1,25D is known to increase VDR transcript levels and protein stability [36]. The levels of VDR and DNA repair factors in HGPS cells were monitored by western blot prior to entry into senescence, and compared to those of NF of similar passage. We discovered that HGPS cells grown in normal media exhibit lower levels of 53BP1 and BRCA1 than NF, in addition to the decrease in VDR levels (Figure 2A and 2B). Prolonged treatment of HGPS cells with 1,25D increased VDR expression, as well as 53BP1 and BRCA1 levels (Figure 2A and 2C). Importantly, prolonged 1,25D treatment markedly reduces progerin protein levels (Figure 2A and 2C). Immunofluorescence analysis confirmed the substantially reduced progerin expression in HGPS cells treated with 1,25D (Figure 2D), when compared to vehicle-treated controls. Moreover, analysis of transcript levels from the *LMNA* gene by qRT-PCR revealed that prolonged 1,25D treatment reduces both total *LMNA* and progerin transcript levels (Figure 2E). These results indicate that 1,25D treatment has multiple beneficial effects in HGPS cells; stabilizing VDR and DNA repair factors, while reducing progerin expression. Of note, we did not detect any differences in the subcellular localization of VDR between NF and HGPS fibroblasts treated with 1,25D or vehicle control (Figure S1).

Next, we investigated potential mechanisms behind the rescue of DNA repair factors (53BP1 and BRCA1) upon 1,25D treatment of HGPS cells. Our previous studies in lamin A/C-depleted cells demonstrated activation of cathepsin L (CTSL)-mediated degradation of 53BP1. We also showed that 1,25D and the broad cathepsin inhibitor E-64 inhibit CTSL activity, leading to stabilization of 53BP1 in lamin A/C-depleted cells [37]. Here, we determined if CTSL could be involved in the decrease of 53BP1 in HGPS cells. A second line of HGPS cells under prolonged treatment with 1,25D or vehicle (Control) was subjected to acute treatment with E-64 for 24 h (Figure 2F). As expected, control HGPS cells exhibit low levels of 53BP1, BRCA1, and VDR, which are stabilized by 1,25D treatment. Interestingly, E-64 treatment restores the levels of 53BP1 in HGPS control cells, suggesting that the same mechanism of 53BP1 degradation discovered in lamin A/C-depleted cells is active in progerin-expressing HGPS cells. In contrast, E-64 was unable to rescue the levels of BRCA1 or VDR, indicating that the loss of these factors in HGPS cells does not involve CTSL. These studies reveal a mechanism behind the loss of 53BP1 in HGPS cells. Importantly, 1,25D treatment reduces significantly the accumulation of DNA damage in HGPS cells, as shown by a marked decrease in the percentage of γH2AX-positive cells (Figure 2G and 2H), and a reduction in average intensity of labeling with γH2AX in 1,25D-treated HGPS cells (Figure 2I), when compared to vehicle-treated cells. Our data demonstrate that VDR deficiency contributes to DNA repair defects in HGPS patient cells and that 1,25D treatment rescues expression of VDR and key DNA repair factors. Vitamin D-based regimens could represent a strategy to restore DNA repair capabilities in HGPS cells, a major cause of the pathology associated with this disease.

### 1,25D reduces nuclear defects and delays senescence

**Figure 3:**
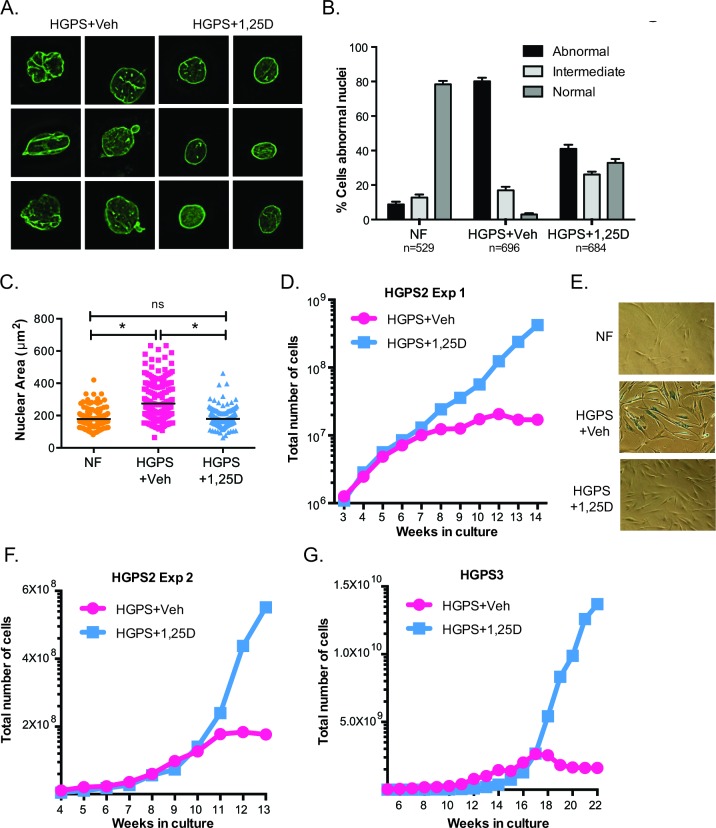
Vitamin D rescues nuclear abnormalities and delays senescence in HGPS cells **A.** DAPI staining and IF with lamin A antibody performed in NF and HGPS fibroblasts under prolonged treatment with vehicle or 1,25D (passage 25). Representative images are shown. **B.** Quantitation of percentage of cells showing aberrant nuclear morphology (extensive protrusions, lobulations, herniations, etc), an intermediate phenotype (slight change in nuclear morphology), and normal nuclear morphology. More than 500 cells were counted per condition in 3 independent blinded experiments. **C.** The nuclear volume of cells processed for IF as in (A) was calculated using Leica's microscope software. A total of 200 cells were quantitated. **D.** Proliferation rate monitored during culture of HGPS cells in media containing 1,25D or vehicle. Note how vehicle-treated HGPS cells growth arrested after approximately 8-10 weeks in culture while 1,25D-treated cells continued proliferating. **E.** β-galactosidase assay performed in NF and HGPS cells treated with 1,25D or vehicle, once vehicle-treated cells growth arrested (passage 33). **F.** Proliferation rate of a second long-term treatment of the same line of HGPS fibroblasts with 1,25D or vehicle control. **G.** Proliferation rate of fibroblasts from a different HGPS patient.

In addition to genomic instability, HGPS cells exhibit gross nuclear architectural disruptions and nuclear blebbing as a result of progerin accumulation at the nuclear envelope [6, 38]. The severity of these nuclear aberrations often correlates with the degree of cellular decline. Given the robust effect of 1,25D reducing progerin and DNA damage in HGPS cells, we investigated whether 1,25D improves nuclear morphological abnormalities in these cells. HGPS cells under prolonged treatment with 1,25D or vehicle were processed for immunofluorescence with lamin A antibody and counterstained with DAPI, to visualize the distribution of lamin A in the nucleus (Figure 3A). In normal fibroblasts, a thin layer of lamin A was distributed evenly at the nuclear periphery, highlighting the edge of the nucleus that was ovular in shape. In contrast, HGPS cells had grossly disrupted nuclear morphology and extensive nuclear blebbing. Lamin A/progerin was localized at the nuclear periphery, but was unevenly distributed. Importantly, cells treated with 1,25D showed a significant improvement in nuclear morphology. Analysis of over 500 cells per condition revealed a marked decrease in the percentage of cells with abnormally shaped nuclei (Figure 3B). Moreover, HGPS cells grown in culture exhibit a marked increase in nuclear volume when compared to NF (Figure 3C), and 1,25D treatment reduces the nuclear volume. These data demonstrate that 1,25D treatment improves profoundly nuclear morphology in HGPS patient cells, which in turn has the potential to ameliorate cellular decline.

To test the hypothesis that 1,25D improves the proliferation of HGPS cells in culture, we compared proliferation rates between cells subjected to prolonged treatment with 1,25D (10^−7^M) or vehicle. Initially, HGPS cells treated with 1,25D grew at a slightly slower rate than those treated with vehicle. This was not entirely surprising, since 1,25D has anti-proliferative effects, particularly in cancer cells [39] (Figure S2B). However, with passage in culture, 1,25D-treated HGPS cells began to grow at a higher rate than those treated with vehicle (Figure 3D). Eventually, HGPS cells treated with vehicle became growth arrested, and presented characteristics of senescence such as positivity for β-galactosidase (Figure 3D and 3E), while those treated with 1,25D continued proliferating. These results were confirmed a second time in the same HGPS line (Figure 3F), as well as in a different HGPS line (Figure 3G). 1,25D-treated HGPS cells eventually entered senescence, but at much later passages. Thus, prolonged treatment with 1,25D allows HGPS patient cells to delay premature entry into senescence.

In summary, our studies demonstrate that 1,25D treatment of HGPS cells reduces accumulation of DNA damage, improves nuclear morphology, and allows bypass of premature senescence. Thus, the 1,25D/VDR axis represents a novel target for treatment of laminopathies such as HGPS, in which expression of a mutant form of lamin A causes cellular decline and organismal degeneration.

### 1,25D/VDR axis modulates *LMNA* gene expression

**Figure 4:**
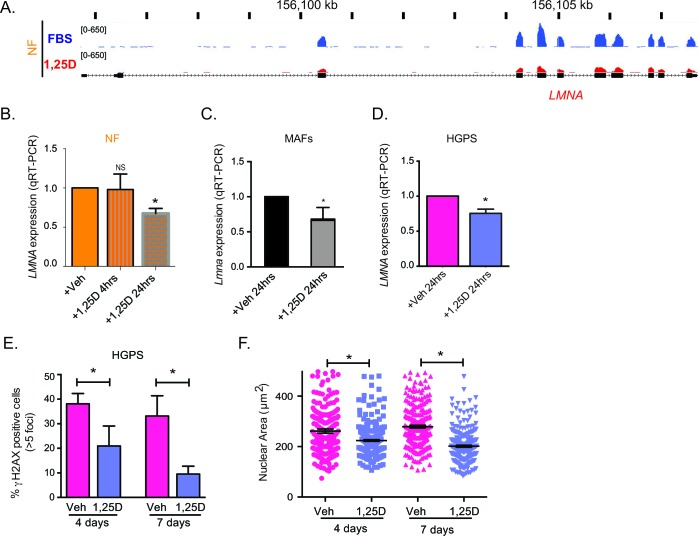
Regulation of LMNA gene expression by vitamin D/VDR **A.** RNAseq analysis performed in NF after a 24-h treatment with 1,25D (10^−7^M) or vehicle (FBS) as control. Map shows the levels of transcripts containing exon sequences of the *LMNA* gene in control cells (blue) and 1,25D-treated cells (red). **B.** Relative expression of total transcripts from *LMNA* gene by qRT-PCR in NF treated with 1,25D for 4 h or 24 h. Results are the mean±sem of 3 biological repeats. **C.** Relative expression of *LMNA* transcripts by qRT-PCR in MAFs treated with 1,25D for 24 h. **D.** Relative expression of total transcripts from *LMNA* gene as determined by qRT-PCR in HGPS patient-derived treated with 1,25D for 24 h or vehicle control. **E.** IF for γH2AX in HGPS cells treated with 1,25D or vehicle for 4 and 7 days. Graph shows percentage of γH2AX-positive cells. A total of 400 cells were quantitated per condition. **F.** DAPI staining and IF with lamin A antibody in HGPS cells treated with 1,25D or vehicle for 4 and 7 days. The nuclear volume was calculated using Leica's microscope software. A total of 300 cells were quantitated. All graphs represent mean±sem. *p value of statistical significance (*p ≤ 0.05).

Given that prolonged 1,25D treatment decreased *LMNA* and progerin expression, we investigated if the *LMNA* gene is directly regulated by the 1,25D/VDR axis, especially because VDR is a nuclear receptor with transcriptional activity. To this end, we tested the effect of short 1,25D treatments on *LMNA* gene expression, and monitored the binding of VDR to the proximity of the *LMNA* gene by chromatin immunoprecipitation followed by sequencing (ChIP-seq). First, genome-wide RNAseq analysis of NF treated with 1,25D (10^−7^M) or vehicle for 24 hours, revealed a decrease in total *LMNA* transcript levels after 1,25D treatment (Figure 4A). In addition, qRT-PCR performed in NF after a 4-hour and a 24-hour treatment with 1,25D confirmed a modest but consistent decrease in *LMNA* transcripts at 24, but not at 4 hours (Figure 4B). The same effect of 1,25D on *LMNA* gene expression was observed in BJ fibroblasts (Figure S2C) and in mouse cells. Mouse adult fibroblasts (MAFs) isolated from the ears, and immortalized with SV40 large T antigen were treated with 1,25D (10^−7^M) for 24 hours. As shown in Figure 4C, 1,25D treatment reduces total *LMNA* transcripts. Similarly, mouse embryonic fibroblasts (MEFs) treated with 1,25D for two weeks exhibit reduced *LMNA* transcripts (Figure S2D). These results demonstrate that 1,25D treatment can regulate total *LMNA* transcript levels in human and mouse cells.

Importantly, ChIP-seq analysis on hematopoietic cell lines and BJ fibroblasts treated with 1,25D or vehicle for up to 24 hours did not show VDR binding to any genomic region near the *LMNA* gene transcription start site (TSS) under any condition (Figure S2). In summary, ChIP-seq, RNA-seq and qRT-PCR analyses suggest that the effect of 1,25D/VDR regulating *LMNA* gene expression is likely to be indirect, putatively by facilitating the function of unknown transcription factors being the main regulators of the *LMNA* gene. Upon ligand activation, VDR decreases *LMNA* gene transcripts, possibly by counteracting the function of the factor/s regulating *LMNA* gene expression [40]. Although further studies are required to identify the VDR-regulated factors mediating *LMNA* gene expression, our data provide strong evidence for the use of 1,25D to reduce expression of mutant lamins.

Lastly, we monitored the effect of short treatments with 1,25D on *LMNA* gene expression in HGPS cells. As shown in Figure 4D, total *LMNA* transcripts are decreased after a 24-hour treatment. Importantly, 1,25D treatment of HGPS cells for 4 and 7 days ameliorates some of the phenotypic abnormalities of HGPS cells. In particular, the short treatment progressively ameliorates the accumulation of DNA damage (Figure 4E), and reduces nuclear volume (Figure 4F). The effect of 1,25D on nuclear size is specific of HGPS cells, as NF did not exhibit any changes in nuclear size upon 1,25D treatment (data not shown). These results indicate that while the mechanism whereby 1,25D down-regulates progerin expression is likely indirect, 1,25D/VDR signaling has a significant beneficial effect on HGPS cellular phenotypes, which is noticeable a few days after treatment.

## DISCUSSION

In laminopathies such as HGPS, the nuclear lamina is severely disrupted, causing alterations in the activities of various transcription factors [35, 41, 42]. Here, we show that depletion of lamin A/C or accumulation of progerin results in decreased expression of VDR, a nuclear receptor that regulates approximately 3% of the transcriptome and mediates most genomic actions of vitamin D [20, 40]. Alterations in lamin A/C expression result in mislocalization of genes in the 3D nuclear space, which in turn impact on their transcriptional regulation. Thus, nuclear localization of the VDR gene could be altered in HGPS cells. Alternatively, progerin could impact the epigenetic status of the VDR gene, leading to repression. Moreover, since VDR regulates its own expression, a direct association of lamin A/C with VDR protein could potentially regulate VDR stability and/or transcriptional activity. Future studies need to identify the mechanisms behind VDR loss in cells with an altered nuclear lamina.

**Figure 5:**
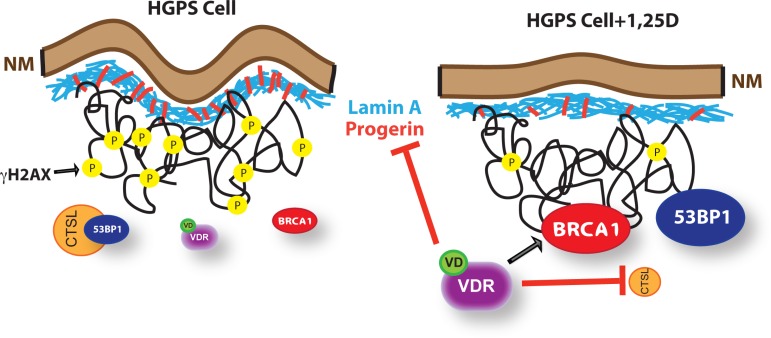
Model of functional relationship between nuclear lamina integrity, vitamin D/VDR axis, and expression of LMNA gene We propose a model whereby in cells with an integral nuclear lamina, expression of DNA repair factors such as BRCA1 and 53BP1, and DNA repair capabilities are maintained by the vitamin D/VDR signaling axis. As such, depletion of VDR in normal fibroblasts results in BRCA1 loss, accumulation of DNA damage, and premature senescence. Cells with a disrupted nuclear lamina due to lamin A/C depletion or progerin accumulation (HGPS) experience a marked reduction in VDR levels, which in turn contributes to the down-regulation of BRCA1 and the activation of CTSL-mediated degradation of 53BP1. These cells accumulate DNA damage markers (γH2AX), nuclear morphological abnormalities and other cellular alterations that ultimately cause premature senescence. Importantly, treatment of HGPS cells with 1,25D to stabilize and activate VDR results in reduced progerin production, stabilization of BRCA1, inhibition of CTSL-mediated degradation of 53BP1, and amelioration of a variety of nuclear phenotypes. This suggests multiple benefits of a vitamin D based therapy for HGPS and other laminopathies.

We also demonstrate that 1,25D/VDR regulates *LMNA* gene expression. 1,25D causes down-regulation of *LMNA* transcripts after 24 hours, suggesting that the effect is indirect. This is supported by the lack of VDR binding in the vicinity of the *LMNA* gene TSS, as shown by ChIP-seq analysis. Importantly, we observed a dramatic decrease in progerin levels in cells under prolonged 1,25D treatment, suggesting that 1,25D treatment could have dual beneficial effects for HGPS cells, ameliorating the toxicity associated with progerin expression, while rescuing VDR function (Figure 5).

Deficiency in VDR function has many detrimental effects. For instance, vitamin D/VDR deficiencies are correlated with cardiovascular disease, bone defects, and a whole variety of inflammatory diseases, and vitamin D-deficient individuals have higher risk of developing cardiac hypertrophy, hypertension, and myocardial infarction [22, 43, 44]. In addition, VDR knockout mice exhibit phenotypes observed in HGPS patients, such as premature aging, atherosclerosis, and cardiovascular disease (CVD) [24, 45], suggesting that impaired VDR activity could contribute to the pathophysiology of HGPS. In fact, we demonstrate that prolonged treatment of HGPS patient-derived fibroblasts with 1,25D rescues DNA repair defects, ameliorates nuclear morphological abnormalities, and allows senescence bypass. Based on these data, and the reported roles of vitamin D in cardiovascular protection [46], we propose that sufficient vitamin D supplementation could benefit HGPS patients.

The finding that vitamin D reduces progerin levels is of high significance (Figure 5). Studies have shown a decrease in progerin protein levels by treatment with rapamycin or sulforaphane, by activating its clearance *via* autophagy [47, 48]. In addition, treatment with retinoids down-regulate total *LMNA* gene expression and decrease progerin expression in HGPS cells [49–51]. This effect is mediated by the retinoic acid receptor (RAR), which binds to regulatory elements in the *LMNA* gene promoter. Future studies need to test if the combination of vitamin D with rapamycin or sulforaphane, or with retinoids has the most benefit as a treatment for HGPS, by reducing progerin transcripts and increasing progerin protein clearance by autophagy. In addition, since progerin expression is observed in cells from old individuals [19], and VDR deficiency and genomic instability increase with age [52, 53], maintaining vitamin D/VDR signaling could ameliorate pathologies associated with normal aging. However, when deciding on a treatment for HGPS it is important to consider that treatment with 1,25D and retinoids not only reduces progerin expression, but also total transcripts from the *LMNA* gene. There is clear evidence that the amount of progerin relative to prelamin A/lamin A dictates the severity of the disease [54, 55]. In fact, recent studies have shown a dosage-dependent effect of progerin expression in normal fibroblasts inducing phenotypes characteristic of HGPS fibroblasts [56]. Thus, further studies are needed *in vitro* and *in vivo* to determine the 1,25D or retinoid regimens that reduce progerin levels under a threshold while maintaining sufficient levels of prelamin A/lamin A as to reduce phenotype severity.

In addition to the role of vitamin D/VDR regulating *LMNA* gene expression, we present evidence for a role of VDR in the maintenance of DNA repair factors such as BRCA1 and 53BP1, which maintain genome integrity by facilitating DNA double-strand break repair (Figure 5). As such, VDR loss in human primary cells hinders DNA repair capabilities and induces senescence. We also show that HGPS cells exhibit decreased levels of BRCA1 upon passage in culture, which are rescued by prolonged 1,25D treatment, thus suggesting that VDR deficiency contributes to DNA repair defects in HGPS cells. We envision that vitamin D/VDR deficiencies could contribute to the genomic instability that drives aging and aging-related diseases. Reduced BRCA1 function in VDR-deficient cells could contribute to cellular decline during aging, while allowing secondary hits in the genome that promote tumorigenesis. Studies that investigate in depth the functional relationship between BRCA1 and the vitamin D/VDR axis could lead to important discoveries about the role of these tumor suppressor pathways in a variety of aging-related diseases.

Numerous studies suggest cardiovascular protection by vitamin D, including anti-atherosclerotic and anti-inflammatory activities [46, 57, 58]. Although our study is the first to demonstrate VDR deficiency in progeria, physicians recommend the intake of 400 IU vitamin D for HGPS children to forestall bone disease (http://www.progeriaresearch.org). We envision that the same VDR reduction that we find in fibroblasts from HGPS patients could extend to other cells, especially vascular cells, and that 400 IU of vitamin D could be insufficient to correct VDR content and biological actions. All these data stress the need for preclinical studies in mouse models of HGPS that explore the benefits of vitamin D-based regimens to ameliorate the progeria phenotypes at the organismal level. Also, the combination of vitamin D regimens with current therapeutic strategies such as FTIs and prenylation inhibitors, could result in increased efficacy of the treatments, and allow lowering the doses of single drugs, reducing toxicity. Importantly, our data suggest that levels of VDR and progerin could be used as biomarkers to screen patients with laminopathies and identify those that could benefit from vitamin D treatment. In addition, monitoring the levels of these proteins before and after vitamin D treatment could serve to determine the effectiveness of the treatment. Thus, vitamin D/VDR signaling holds promise as a target for treatment of HGPS patients, which could ward off the disastrous consequences of this ultimately fatal disease.

## MATERIALS AND METHODS

### Cell culture

Skin fibroblasts from 4 HGPS patients, 2 with classic mutations and 2 with non-classic mutations, and from parents of HGPS patients were obtained from the Progeria Research Foundation (see SI). Cells were maintained in DMEM, 10% FBS, antibiotics/antimycotics. VSMC were obtained from Cell Applications and cultured in Lonza SmBM media with 5% FBS and LonzaSmGM^TM^-2 SingleQuots.

#### Vitamin D treatment

For long-term treatment, cells were supplemented with 10^−7^ M 1,25D (1α,25-dihydroxyvitamin D_3_) every 3 days, as described [34]. Short-term treatments were performed with 10^−7^ M 1,25D for 24 h, 4 days, and 7 days.

#### E-64 treatment

HGPS cells were incubated with the broad-spectrum cathepsin inhibitor E-64 10 μM for 24 h.

### Immunoblotting

Cells were lysed in RIPA buffer (150mM NaCl, 50mM Tris-HCl pH 7.4, 1% NP-40, 0.2% SDS, 0.25% sodium deoxycholate, and 1mM EDTA), containing HALT protease and phosphatase inhibitor cocktail. Lysates were sheared using 10 passes through a 26-gauge needle followed by 10 passes through a 30-gauge needle. 60-120μg of total protein was loaded on 4-15% Criterion TGX Gel. List of antibodies used in SI.

#### Quantitative reverse-transcription PCR

cDNA was generated from 1μg total RNA using the GeneAmp^®^ RNA PCR kit. qRT-PCR was performed using the 7500HT Fast Real-Time PCR system with the Taqman^®^ Universal PCR Master Mix or Universal SYBR Green Supermix. Reactions were carried out in triplicate and target gene and endogenous controls were amplified in the same plate. Relative quantitative measurements of target genes were determined by comparing the cycle thresholds. List of probes used in SI.

### Immunofluorescence

IF, microscopy and photo capture were performed exactly as described [34]. Cells were considered positive for γH2AX when exhibiting more than 5 nuclear foci. Staining intensity for γH2AX was quantitated using ImageJ. DAPI staining was used to select nuclear area, and mean signal intensity was then determined on the γH2AX staining. Background signal was accounted for and subtracted from mean signal intensity. Lamin A pictures were taken using 3D deconvolution in the Leica Application Suite. Cell morphology was then scored as normal or abnormal based on blebbing or rupture of the nucleus. Progerin staining intensity and nuclear area were quantitated from the progerin and DAPI pictures using the Leica Application Suite.

### Viral transduction

Lentiviral transductions were performed as described [59]. Different shRNAs were purchased from Sigma-Aldrich, except for the progerin plasmid, which was a gift from Brian Kennedy (Buck Institute for Research on Aging, Novato, CA).

### Proliferation assays

Cells were plated in triplicate at 600,000 cells/10-cm plate, and counted when confluency was near 80% utilizing Trypan Blue Viability Assay on the Nexcelom Cellometer Vision CBL. To extrapolate proliferation to the respective time periods, we used the equation N_f_ = N_0_e^kt^, as in [34].

### Senescence-associated β-galactosidase staining

Cells were fixed in 0.2% glutaraldehyde for 5 min at 37°C, washed in PBS, and incubated for 24 h in fresh senescence β-galactosidase staining solution (1 mg 5-bromo-4-chloro3-indolyl β-D-galactoside per mL of buffer containing: 40 mM sodium phosphate/150 mM NaCl/5 mM potassium ferrocyanide/5 mM potassium ferricyanide/2 mM MgCl_2_ at pH 6). After 4-6 h incubation at 37°C, cells were washed in PBS and pictures taken.

### Analysis of cell cycle profile

Cells were fixed in ice-cold 70% ethanol while vortexing and stored at 4°C until analysis. Fixed cells were washed with PBS, suspended in 1 ml propidium iodide staining (400 μg /ml), and incubated for 15 min at 37°C. Stained cells were analyzed for DNA content using the Nexcelom Cellometer Vision CBL, and cell cycle profiles were created by the program FCS Express 4 Flow Cytometry.

### Statistical analysis

For all qRT-PCR experiments, a standard “two-tailed” student's *t*-test was used to calculate statistical significance of the observed differences. Microsoft Excel v.2010 was used for the calculations. For γH2AX and progerin labeling experiments a one-way ANOVA test was performed using GraphPad Prism software. In all cases, differences were considered statistically significant when *p* < 0.05.

### RNAseq and ChIPseq described in supplementary methods

## SUPPLEMENTARY FIGURES


