# Supplementary material for: Vitamin D receptor signaling improves Hutchinson-Gilford progeria syndrome cellular phenotypes
Source: Oncotarget. 2016 Apr 27;7(21):30018–31. doi: 10.18632/oncotarget.9065 (PMC5058660; doi:10.18632/oncotarget.9065)
Supplement: Supplementary file 1 [file oncotarget-07-30018-s001.pdf]

Vitamin D receptor signaling improves Hutchinson-gilford progeria syndrome cellular phenotypes

Supplementary Information

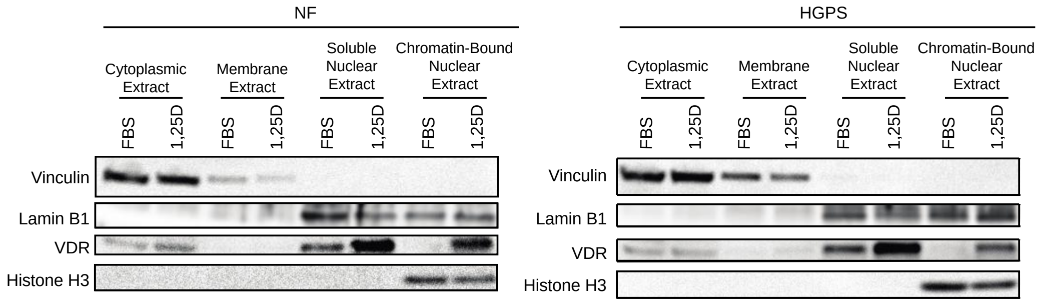

Supplementary Figure 1. Subcellular fractionation to monitor VDR localization. Subcellular fractionation was performed to compare the localization of VDR between NF and HGPS fibroblasts. Vinculin was used as a marker of the cytoplasmic fraction, lamin B1 as a marker of the nuclear fraction, and histone H3 as a marker of the chromatin fraction. Note how VDR is found primarily in the soluble nuclear fraction in both NF and HGPS cells and how 1.25D treatment increases the amount of VDR in the nuclear fractions. No significant differences in subcellular distribution of VDR were observed between NF and HGPS cells. Of note, the amount of HGPS cells utilized for subcellular fractionation was higher than that of NF in order to detect VDR protein.

A.

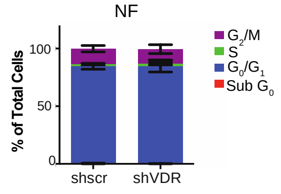

B.

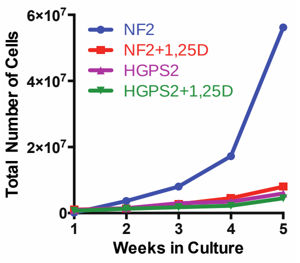

NF	shscr	shVDR
$SubG_1$	0.4%	0.3%
G_0/G_1	84.4%	84.8%
S	1.5%	2.0%
G_2/M	13.7%	12.9%

C. D.

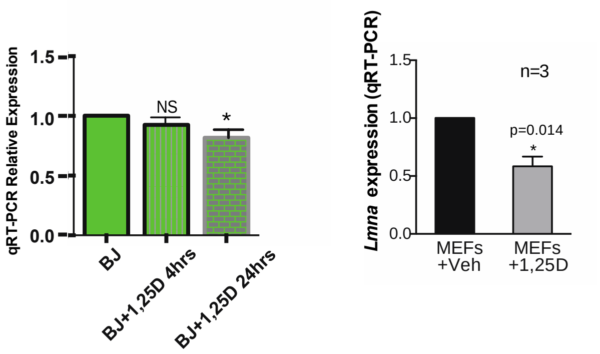

Supplementary Figure 2. Effects of changes in VDR activity in cell cycle and *LMNA* gene expression, and proliferation. (**A**) NF lentivirally transduced with a shRNA targeting VDR (shVDR) or a shRNA control (shscr) were collected after selection with puromycin. Cells were processed for immunoblotting (Fig 1F), and fixed for cell cycle profile analysis by staining with propidium iodide. DNA content was analyzed using a Nexcelom Cellometer. Note how acute depletion of VDR does not cause alterations in the cell cycle. However, these cells will undergo growth arrest after 2 weeks in culture (see main manuscript). (**B**) Proliferation of NF and HGPS cells in the first weeks after 1,25D addition. Note the differences in proliferation between NF and HGPS cells, and how 1,25D slows down the proliferation of NF, and to a lesser extent of HGPS cells, consistent with its anti-proliferative effects. 1,25D-treated NF will continue proliferating with no signs of distress for extended periods of time in culture. In HGPS cells, 1,25D treatment will improve their phenotype allowing them to delay senescence. (**C**) Relative expression of *LMNA* transcripts by qRT-PCR in BJ fibroblasts treated with 1,25D for 4 or 24 hours. Results are the mean±sem of 3 biological repeats. (**D**) Relative expression of *LMNA* transcripts by qRT-PCR in MEFs treated with 1,25D for 2 weeks. Results are the mean±sem of 3 biological repeats.

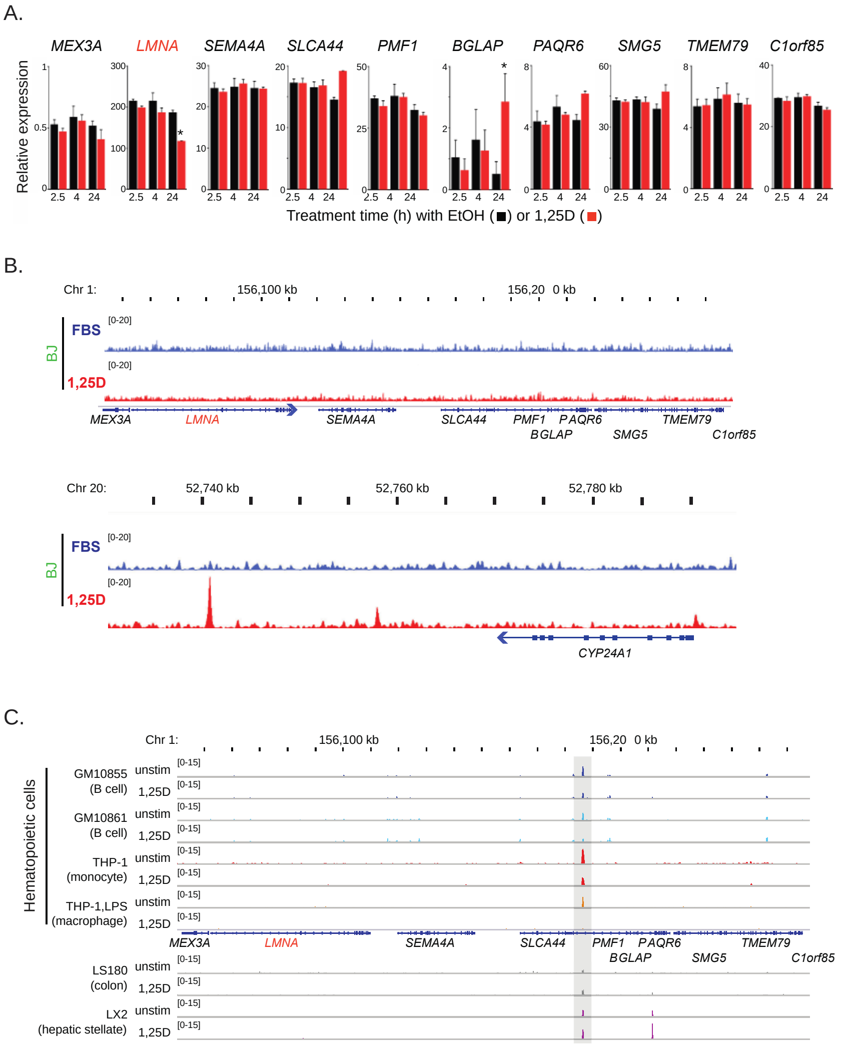

Supplementary Figure 3. ChIPseq analysis of VDR binding in the *LMNA* gene locus. (**A**) RNA-seq data from THP-1 cells treated for 2.5, 4, and 24 h with 10^{-7}M 1,25D indicated significant down-regulation of the *LMNA* gene after 24 h ligand treatment and up-regulation of the neighboring *BGALP* gene [1]. RNA-seq raw data are available at Gene Expression Omnibus (http://www.ncbi.nlm.nih.gov/geo/). (**B**) ChIP with VDR antibody and IgG as control was performed in BJ fibroblasts treated with 10^{-7}M 1,25D or vehicle (FBS) for 24 h. ChIP-seq libraries for input chromatin, VDR ChIP no treatment, and VDR ChIP 1,25 D treatment were constructed and sequenced. The IGV browser was used to monitor VDR occupancy at the *LMNA* gene locus. We did not find VDR binding in the TSS or any other region of the *LMNA* gene locus. In contrast, VDR binding was observed in the vicinity of the CYP24A1 locus, a known VDR-regulated gene. (**C**) The IGV browser was used to monitor human VDR ChIP-seq data [2] at the *LMNA* gene locus in six different human cell lines treated with 1,25D or unstimulated. We did not find binding of VDR near the TSS. However, we did find a conserved VDR binding site located 130 kb downstream of the *LMNA* TSS. In hematopoietic cells, VDR occupancy at this site is reduced after 1,25D treatment.

Supplementary Methods

Cells from Progeria Research Foundation: Skin fibroblasts from 2 HGPS patients with classic mutations (HGPS2 and HGPS9) and 2 with non-classic mutations (HGPS3 and HGPS5), and from parents of HGPS patients (PSFDFN327; HGMDFN090; PSMDFN346) were obtained from the Progeria Research Foundation. Description of mutant HGPS lines: HGPS2; HGADFN167 from an 8 yo male, *LMNA* exon 11 heterozygous c.1824C>T (p.Gly608Gly). HGPS9; HGADFN003 from a 2 yo male, *LMNA* exon 11 heterozygous c.1824C>T (p.Gly608Gly). HGPS3; PSADFN392 from a 7 yo male, *LMNA* exon 11 c.1968+2T>C. HGPS5; PSADFN257 from a 1 yo male, *LMNA* exon 10, homozygous c.1619 T>C (p.Met540Thr).

***List of antibodies used for immunoblotting*:** γH2AX (#07-164, Millipore or #2577, Cell Signaling); Lamin A/C (sc-20681, SCBT); Progerin (SAB4200272, Sigma-Aldrich); Vinculin (sc-7649, SCBT); VDR (sc-13133, SCBT); 53BP1 (NB100-304 Novus Biological or H-300: sc-22760 SCBT); BRCA1 (OP93, Millipore); β-Tubulin (T8238, Sigma Aldrich); Lamin A (ab26300, Abcam).

***List of probes used for qRT-PCR*:** *VDR* Hs01045840_m1 and Hs0172113_m1; *LMNA* Hs0015346_m1; *18S* human Hs99999901_s1; *Gapdh* mouse Mm99999915_g1; *Lmna* Mm00497783_m1. SYBR Green Primers: Human *LMNA* F–GAGGAGGGCAAGTTTGTCCG / R–CATTCTGGCGCTTGATCTGC; Human *GAPDH* F-GCATGGCCTTCGGTGTCC / R-AATGCCAGCCCCAGCGTCAAA; Human Progerin F-GCGTCAGGAGCCCTGAGC / R-GACGCAGGAAGCCTCCAC; Human Lamin A only F-GGACAATCTGGTCACCCGC / R-GCGTCAGGAGCCCTGAGC; Mm *Gapdh* F-

GTGCAGTGCCAGCCTCGTCC / R-GCCACTGCAAATGGCAGCCC; Mm *Lmna* F-ACCCCGCTGAGTACAACCT / R-TTCGAGTGACTGTGACACTGG

Antibodies used for immunofluorescence: γH2AX (#07-164, Millipore or #2577, Cell Signaling); Lamin A (ab26300, Abcam); Progerin (SAB4200272, Sigma-Aldrich); Goat anti-Mouse and Goat anti-Rabbit Alexa Fluor 594/488-labeled secondary antibodies (Invitrogen).

RNAseq

Total RNA was isolated from NF supplemented with 10^{-7} M 1,25D (1α,25-dihydroxyvitamin D_3; Sigma-Aldrich) or FBS for 24 h using Aurum Total RNA Mini Kit (Bio-Rad) with DNaseI treatment following the manufacturer's protocol. Ribosomal RNA depletion was performed with 500 ng of total RNA with the Ribominus Eukaryotic System v2 (Life Technologies) according to the manufacturer's directions. The rRNA-depleted RNA was used to construct barcoded libraries using the Ion Total RNA-Seq Kit v2 (Life Technologies), following the manufacturer's instructions. The libraries were sequenced on an Ion Torrent Proton with a mean read length of 103 nucleotides. RNA-seq quantification was performed using a method similar to that described described before [3]. Sequencing reads were aligned to the hg19 genome sequence using the map4 algorithm in the TMAP aligner (https://github.com/iontorrent/TMAP/blob/master/doc/tmap-book.pdf), allowing soft clipping of both the 5' and 3' ends (option -g 0) and a minimum seed length of 20. The resulting bam files were sorted using SAMtools [4] and used to generate strand-specific bedgraph files of the base pair coverage using BEDTools [5]. A custom R (R Core Team; http://www.R-project.org) script was used to convert the strand-specific bedgraph

files to sgr files, which were used to sum the strand-specific base pair coverage of non-redundant sets of plus strand and minus strand exons using a custom R script. The nucleotide coverage values of all exons for each gene were summed using an R script to generate nucleotide coverage values for all annotated genes. The coverage values for each gene were normalized to the total coverage for all genes for each library to allow quantitative comparisons.

ChIPseq

BJ fibroblasts at approximately 80% confluency were treated with 10^{-7} M 1,25D or FBS (vehicle) for 24 h, and cells crosslinked by addition of 36% formaldehyde directly to the cell culture media to a final concentration of 1%. After incubation for 10 minutes at room temperature on a rocking platform, crosslinking was stopped by addition of glycine pH 7.0 to a final concentration of 0.125 M. Cells were washed twice with cold PBS, scraped and lysed at a density of $5X10^6$ cells ml^{-1} for 10 min at 4°C in 1% SDS, 50 mM Tris-HCl (pH 8.0) and 10 mM EDTA containing protease inhibitors. Lysates were sonicated to obtain chromatin fragments <1 Kb and centrifuged for 15 min in a microfuge at RT. Lysates were diluted 1:10 with 1.1% Triton-X100, 2 mM EDTA, 150 mM NaCl and 20 mM Tris-HCl (pH 8.0) containing protease inhibitors, and precleared with blocked magnetic beads (protein-G-Dynabeads blocked in 1X PBS+0.5% BSA and resuspended in dilution buffer). Fragments were incubated with 10 μg of VDR antibody or 10 μg of control IgG, together with 80 μl of Dynabeads mix at 4°C overnight on a rotating platform. Next day, immunoprecipitates were washed with 0.1% SDS, 1% Triton-X100, 2 mM EDTA, 20 mM Tris-HCl (pH 8.0) and 150 mM NaCl (one wash); 0.1% SDS, 1% Triton-X100, 2 mM EDTA, 20 mM Tris-HCl (pH 8.0) and 500 mM NaCl (one wash); 0.25

M LiCl, 1% Nonidet P-40, 1% sodium deoxycholate, 1 mM EDTA and 10 mM Tris-HCl, pH 8.0 (one wash); and 10 mM Tris-HCl (pH 8.0) and 1 mM EDTA (two washes). Chromatin was eluted from the beads twice by incubating with 250 μl 1% SDS and 0.1 M $NaHCO_3$ during 15 min at RT with rotation. After adding 20 □l of 5 M NaCl, we reversed the crosslinks for 4 h at 65 °C. Samples were supplemented with 20 μl of 1 M Tris-HCl (pH 6.5), 10 μl of 0.5 M EDTA, 20 μg of RNase A and 40 μg of proteinase K and incubated for 1 h at 45 °C. We recovered DNA by phenol-chloroform extraction and ethanol precipitation. For Input samples, we collected 20 μl of chromatin after sonication, and processed them with the rest of the samples at the point of reverse crosslinking.

Barcoded ChIP-seq libraries for input chromatin, VDR ChIP no treatment, and VDR ChIP vitamin D treatment were constructed using the Ion Plus Fragment Library Kit (Life Technologies) according to the manufacturer's directions, and sequenced on an Ion Torrent Proton, with a mean read length of 146 nucleotides. Reads were aligned to the hg19 genome sequence (minus all unassigned contigs) using the map4 algorithm in the TMAP aligner without soft-clipping (option –g 3). SAMtools was used to sort the bam files and separate them into individual chromosomes. The bam files from the inputs for the treated and untreated were merged prior to separating into individual chromosomes to increase coverage for normalization. The BEDTools *genomecoveragebed* program was used to generate an sgr file with nucleotide coverage at each nucleotide for each chromosome (option –d). Custom R scripts were used sum the coverage in sliding windows (250 bp windows, 50 bp steps), normalize the ChIP window values to the input chromatin windows, calculate enrichment (ChIP / input), and generate sgr graph files for viewing genome browsers.

References:

1. Seuter S, Neme A and Carlberg C. Epigenome-wide effects of vitamin D and their impact on the transcriptome of human monocytes involve CTCF. Nucleic Acids Res. 2016; 44(9):4090-104. doi: 10.1093/nar/gkv1519.
2. Tuoresmaki P, Vaisanen S, Neme A, Heikkinen S and Carlberg C. Patterns of genome-wide VDR locations. PLoS One. 2014; 9(4):e96105.
3. Wu Y, Gause M, Xu D, Misulovin Z, Schaaf CA, Mosarla RC, Mannino E, Shannon M, Jones E, Shi M, Chen WF, Katz OL, Sehgal A, Jongens TA, Krantz ID and Dorsett D. Drosophila Nipped-B Mutants Model Cornelia de Lange Syndrome in Growth and Behavior. PLoS Genet. 2015; 11(11):e1005655.
4. Li H, Handsaker B, Wysoker A, Fennell T, Ruan J, Homer N, Marth G, Abecasis G, Durbin R and Genome Project Data Processing S. The Sequence Alignment/Map format and SAMtools. Bioinformatics. 2009; 25(16):2078-2079.
5. Quinlan AR and Hall IM. BEDTools: a flexible suite of utilities for comparing genomic features. Bioinformatics. 2010; 26(6):841-842.
